# Differential Impact of the *rpoB* Mutant on Rifampin and Rifabutin Resistance Signatures of *Mycobacterium tuberculosis* Is Revealed Using a Whole-Genome Sequencing Assay

**DOI:** 10.1128/spectrum.00754-22

**Published:** 2022-08-04

**Authors:** Ming-Chih Yu, Ching-Sheng Hung, Chun-Kai Huang, Cheng-Hui Wang, Yu-Chih Liang, Jung-Chun Lin

**Affiliations:** a Division of Pulmonary Medicine, Department of Internal Medicine, Wan Fang Hospital, Taipei Medical University, Taipei, Taiwan; b School of Respiratory Therapy, College of Medicine, Taipei Medical University, Taipei, Taiwan; c Pulmonary Research Center, Wan Fang Hospital, Taipei Medical University, Taipei, Taiwan; d Ph.D. Program in Medical Biotechnology, College of Medical Science and Technology, Taipei Medical University, Taipei, Taiwan; e Department of Laboratory Medicine, Wan Fang Hospital, Taipei Medical University, Taipei, Taiwan; f School of Medical Laboratory Science and Biotechnology, College of Medical Science and Technology, Taipei Medical University, Taipei, Taiwan; University of L’Aquila

**Keywords:** MinION, *Mycobacterium tuberculosis*, rifabutin, rifampin, *rpoB*

## Abstract

Drug resistance in Mycobacterium tuberculosis (MTB) has long been a serious health issue worldwide. Most drug-resistant MTB isolates were identified due to treatment failure or in clinical examinations 3~6 months postinfection. In this study, we propose a whole-genome sequencing (WGS) pipeline via the Nanopore MinION platform to facilitate the efficacy of phenotypic identification of clinical isolates. We used the Nanopore MinION platform to perform WGS of clinical MTB isolates, including susceptible (*n *=* *30) and rifampin- (RIF) or rifabutin (RFB)-resistant isolates (*n *=* *20) according to results of a susceptibility test. Nonsynonymous variants within the *rpoB* gene associated with RIF resistance were identified using the WGS analytical pipeline. In total, 131 variants within the *rpoB* gene in RIF-resistant isolates were identified. The presence of the emergent Asp531Gly or His445Gln was first identified to be associated with the rifampin and rifabutin resistance signatures of clinical isolates. The results of the minimum inhibitory concentration (MIC) test further indicated that the Ser450Leu or the mutant within the rifampin resistance-determining region (RRDR)-associated rifabutin-resistant signature was diminished in the presence of novel mutants, including Phe669Val, Leu206Ile, or Met148Leu, identified in this study.

**IMPORTANCE** Current approaches to diagnose drug-resistant MTB are time-consuming, consequently leading to inefficient intervention or further disease transmission. In this study, we curated lists of coding variants associated with differential rifampin and rifabutin resistant signatures using a single molecule real-time (SMRT) sequencing platform with a shorter hands-on time. Accordingly, the emerging WGS pipeline constitutes a potential platform for efficacious and accurate diagnosis of drug-resistant MTB isolates.

## INTRODUCTION

Infection with Mycobacterium tuberculosis (MTB) results in tuberculosis, which remains a serious health threat, with around 10 million incident cases and over 1 million deaths attributable to this disease in 2019 (1). Epidemiological control of MTB is hampered by increases in drug-resistant MTB isolates. Drug resistance in MTB is largely mono-resistant to either isoniazid (INH) or rifampin (RIF), and around 1.6 million cases have been caused by multidrug-resistant (MDR) MTB, which is characterized by resistance to both INH and RIF (2, 3). Among these cases, RIF resistance was most often reported after receiving a standard first-line treatment regimen (4). Rapid, accurate diagnosis of RIF resistance is critical for clinicians when determining treatment strategies and for subsequently diminishing community transmission of MTB isolates.

Binding of RIF to the RNA polymerase (RNAP) β subunit of MTB ultimately interferes with DNA transcription and subsequently leads to decreases in RNA products (4). MTB gains rifampin resistance primarily through *rpoB* mutations which are mostly present within an 81-bp rifampin resistance-determining region (RRDR, corresponding to codons 426 to 452 in MTB and codons 507 to 533 in E. coli) (5). Substitutions of amino acid in codons 450, 445, and 435 are most frequently characterized among clinical RIF-resistant isolates (6, 7). The Xpert MTB/RIF (Cepheid, Sunnyvale, CA) assay partially met the requirement for global diagnosis of RIF resistance by identifying the mutations within RRDR through a PCR-based approach (8). Nevertheless, the association of V170F and I491F mutations within the rpoB protein with rifampin resistance was recently revealed by the World Health Organization, which has not been characterized using the Xpert MTB/RIF assay (9).

With the advancement of high-throughput sequencing, the results of whole-genome sequencing (WGS) can provide profiles regarding associations of genetic variants with resistance to all anti-MTB agents (10). Nevertheless, the efficiency of amplicon-based next-generation sequencing (NGS) encountered interference due to a high-GC region within the MTB genome (11). In contrast, long-read sequencing platform is an enticing alternative to widely used NGS platforms for the analysis of highly repetitive regions, which are distinct characteristics of the MTB genome (12). The Nanopore long-read sequencer developed by Oxford Nanopore Technologies (ONT) is potentially practicable for clinical application, with its short hands-on time, low cost, portable device, and customized workflow (13). Moreover, continuous improvement in accuracy rates, smaller amounts of input DNA, and faster hands-on times have re-attracted interest in its clinical application for MTB diagnosis (14–16). Here, the Oxford Nanopore Technologies (ONT) long-read sequencing pipeline was deployed for WGS of susceptible and RIF-resistant MTB isolates. Subsequently, the accordance of the susceptibility test, MIC test, and WGS results with the mutant *rpoB* gene were evaluated. The results demonstrated that the WGS pipeline can serve as an auxiliary and potential test for characterizing emerging *rpoB* mutants which are relevant to the RIF-resistant signature of MTB isolates.

## RESULTS

### Drug-resistance profiles of the enrolled MTB isolates.

As shown in Table 1, the drug susceptibility signatures of enrolled MTB isolates were determined using the agar proportion method. Among the enrolled isolates, 20 showed a resistant signature to rifampin, whereas 14 synchronously exhibited a high-resistance signature to rifabutin (RFB).

**TABLE 1:** Drug-resistance profiles of Mycobacterium tuberculosis isolates enrolled in this study^*a*^

Isolate no.	INH (μg/mL)		RIF (μg/mL)		EM (μg/mL)		SM (μg/mL)	
	0.2	1.0	1.0	0.5	5.0	10.0	2.0	10.0
1	R	S	R	R	R	S	R	R
2	R	S	R	R	R	S	S	S
3	R	S	R	R	S	S	S	S
4	R	S	R	R	S	S	S	S
5	R	S	R	S	S	S	R	R
6	R	S	R	R	R	S	S	S
7	R	S	R	R	S	S	S	S
8	R	R	R	S	R	S	R	S
9	R	Res	R	S	R	S	R	R
10	R	R	R	R	S	S	S	S
11	R	R	R	R	R	S	R	R
12	R	R	R	R	R	S	R	S
13	R	R	R	R	S	S	S	S
14	R	R	R	R	R	S	S	S
15	R	R	R	S	R	S	R	R
16	R	R	R	R	S	S	S	S
17	R	S	R	R	R	Sus	S	S
18	R	R	R	R	R	R	R	S
19	R	R	R	R	R	S	S	S
20	R	S	R	S	S	S	S	S

a INH, isoniazid; RIF, rifampin; RFB, rifabutin; R, resistant; S, susceptible; EM, ethambutol; SM, streptomycin.

### Statistical analysis of long-read sequencing results.

**FIG 1:**
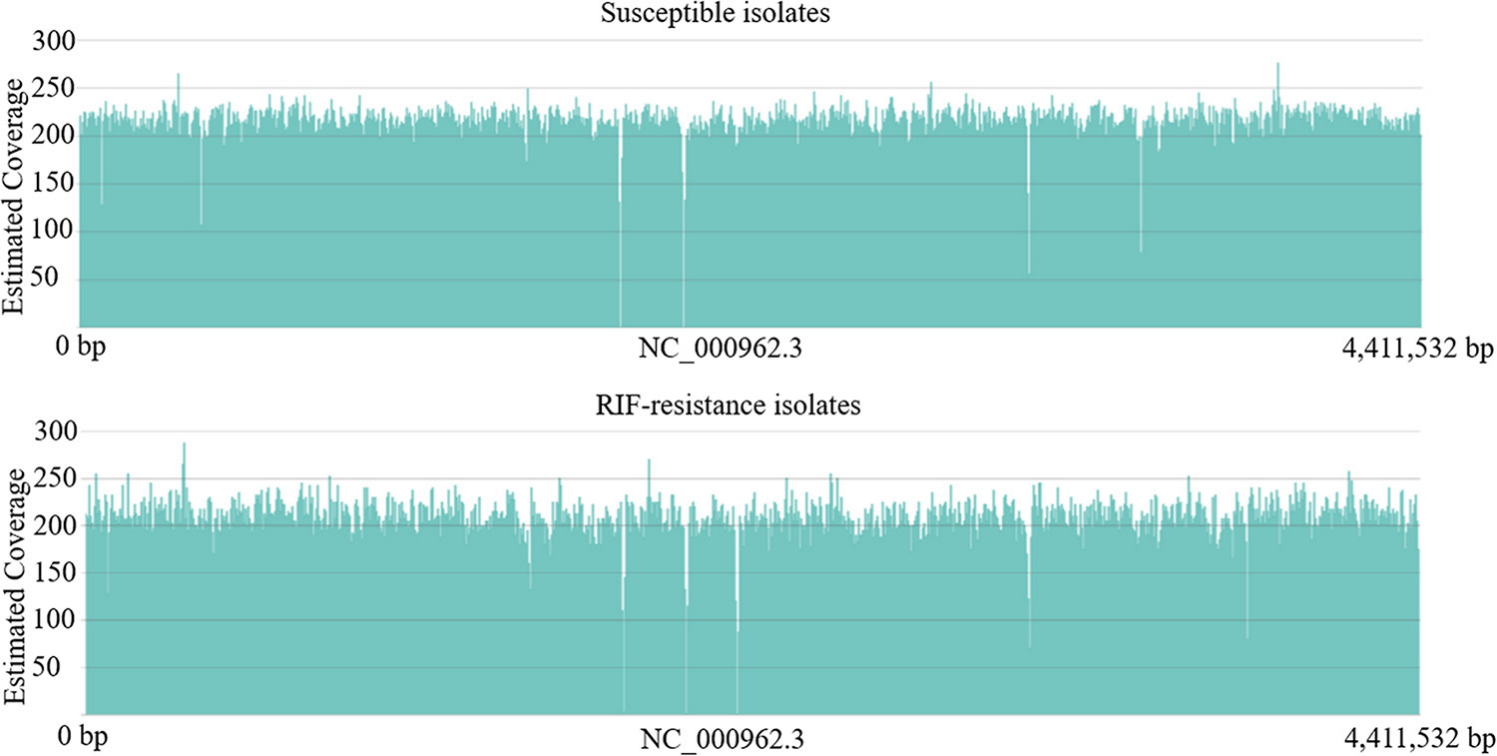
Diagram presenting coverage rates of sequenced reads aligned to the entire Mycobacterium tuberculosis (MTB) genome in each group.

The high-molecular-weight genomic DNA (gDNA) extracted from MTB isolates was subjected to the MinION (ONT) long-read sequencing platform. More than 4 × 10^5^ raw reads per sample were generated from the MinION sequencer in this study. Alignment of the filtered reads to the MTB reference genome (H37Rv, GenBank ID: NC_000962.3) was synchronously conducted using the CLC Genomics Workbench (Qiagen) and the EPI2ME desktop agent algorithm (ONT). No statistical differences in sequencing or alignment efficiencies were noted between the two groups (Table 2, *P* > 0.5). The alignment results with sequenced reads generated using gDNA extracted from the susceptible or RIF-resistant isolates showed over 200× coverage depth toward the full MTB reference genome (Fig. 1). A low coverage for several highly repeated or homo-polymeric regions was noted with the alignment toward the MTB reference genome (Fig. 1).

**TABLE 2:** Statistical results of ONT sequencing in each group^*a*^

Characteristic	Isolate group		*P* value
	Susceptible (*n* = 30)	Rifampin-resistant (*n* = 20)	
No. raw reads, mean (SD)	451,196 (±23,569)	433,651 (±25,342)	>0.5
No. aligned reads, mean (SD)	370,845 (±13,558)	362,517 (±11,459)	>0.5
%Correctly classified (SD)	82.19 (±7.41)	83.59 (±6.47)	>0.5

a SD, standard deviation.

### Identification of nonsynonymous variants within the *rpoB* gene in RIF-resistant isolates.

Clinical MTB isolates with a mutant *rpoB* gene strongly gained a resistance signature to RIF treatment (6). With depletion of synonymous single-nucleotide polymorphisms (SNPs) identified in the enrolled isolates, 230 nonsynonymous variants within the *rpoB* gene were solely characterized in 30 susceptible isolates (Table 3, upper row). Out of 230 amino acid substitutions, 6 were identified within the RRDR region of *rpoB* gene prepared from the susceptible isolates (Table 3, upper row). Among the variants, Asp435Asn and Phe433Ser were novel RRDR mutants identified in this study (Table 3, upper row) (17). In addition, 131 nonsynonymous variants within the *rpoB* gene were solely characterized in RIF-resistant isolates, and nine of 131 amino acid substitutions were identified within the RRDR region (Table 3, lower row). According to the WHO report, Leu443Trp and Leu430Gln variants were first characterized in this study, whereas the presence of the other RRDR mutants was associated with the RIF-resistant signature (17).

**TABLE 3:** List of identified variants within the *rpoB* gene using the ONT sequencing pipeline for RIF-susceptible and RIF-resistant MTB isolates^*a*^

Isolate group	Total, *n*	Nonsynonymous variants within *rpoB*		
		Total, *n*	In RRDR	
			*N*	Types (no. identified copies)
Susceptible	30	230	6	Ser431Arg (5)/Asp435Asn (1)/Thr444Ile (1)/Phe433Ser (1)/Ser428Arg (1)/Thr427Ser (1)
RIF-resistant	20	131	9	Ser450Leu (13)/Ser450Trp (5)/His445Gln (2)/Leu443Trp (2)/His445Leu (1)/His445Tyr (1)/Gln432Lys (1)/Ser431 Arg (1)/Leu430Gln (1)

a ONT, Oxford Nanopore Technologies; RIF, rifampin; MTB, Mycobacterium tuberculosis; RRDR, rifampin resistance-determining region.

### Correlation of genotyping profile with phenotyping results of RIF- and RFB-resistance signatures.

Ser450Leu (S531L in E. coli) is a widely characterized variant within the RRDR region of the *rpoB* gene, which has been closely associated with RIF resistance in MTB (18). In this study, an MIC test was conducted to verify the correlation of genotyping profiles of the mutant *rpoB* gene with RIF-resistance signatures of clinical MTB isolates. The presence of Ser450Leu or Ser450Trp within the *rpoB* gene was associated with significant RIF resistance (>16 μg/mL) and RFB resistance (16 μg/mL) (Table 4, genotyping no. 1, 2, 5, and 6). As reported by the WHO in 2021, Ser450Leu and Ser450Trp are classified as group-1 mutations which exhibit high sensitivity for predicting RIF susceptibility in MTB (17). The clinical isolates harboring the His445Gln or Asp531Gly variants outside the RRDR exhibited significant RIF resistance of >16 μg/mL and high RFB resistance of >8 μg/mL (Table 4, genotyping no. 3 and 4). Correlations of RIF- or RFB-resistance signatures of MTB isolates with the presence of His445Gln or Asp531Gly variants within the *rpoB* gene were first identified in this study. The presence of a novel Leu206Pro variant within the *rpoB* gene exhibited no effect on diminishing the RIF- or RFB-resistant signatures of clinical isolates (Table 4, genotyping no. 5 and 6). In contrast, the presence of the novel Met148Leu, Leu206Ile, and Phe669Val variants outside the RRDR, or the Leu443Trp mutation, were associated with decreased RFB-resistance activities in the group-1 Ser450Leu-, Ser450Trp-, or Gln432Lys-containing isolates (Table 4, genotyping no. 7~10; RFB MIC < 0.5 μg/mL), whereas the RIF-resistance activity was sustained (Table 4, genotyping no. 7~10; RIF MIC of 8 or >16 μg/mL). These results indicated the differential impacts of emerging variants within the *rpoB* gene on RIF- and RFB-resistance signatures.

**TABLE 4:** Profiling results of DST, MIC, and nonsynonymous variants within the *rpoB* gene of drug-resistant MTB^*a*^

Genotyping no.	Variants		MIC (μg/mL)		DST (μg/mL)		Frequency (%)
	High-confidence	Novel	Rifampin	Rifabutin	Rifampin	Rifabutin	
1	Ser450Leu	NA	>16	>16	1	0.5	25% (5/20)
2	Ser450Trp	NA	>16	16	1	0.5	10% (2/20)
3	His445Gln	NA	>16	8	1	0.5	10% (2/20)
4	NA	Asp531Gly	>16	16	1	0.5	10% (2/20)
5	Ser450Leu	Leu206Pro	>16	16	1	0.5	10% (2/20)
6	Ser450Trp	Leu206Pro	>16	16	1	0.5	5% (1/20)
7	Gln432Lys	Phe669Val	>16	0.5	1	0.5	5% (1/20)
8	Ser450Leu	Phe669Val Leu443Trp	>16	0.5	1	0.5	10% (2/20)
9	Ser450Leu	Met148Leu Leu206Ile	>16	0.25	1	0.5	10% (2/20)
10	Ser450Trp	Leu206Ile	>16	0.5	1	0.5	5% (1/20)

a DST, drug-susceptibility test; MIC, minimum inhibitory concentration; MTB, Mycobacterium tuberculosis; NA, not applicable.

### Predictive values of identified variants toward RIF- and RFB-resistance signatures evaluated using an ROC curve analysis.

**FIG 2:**
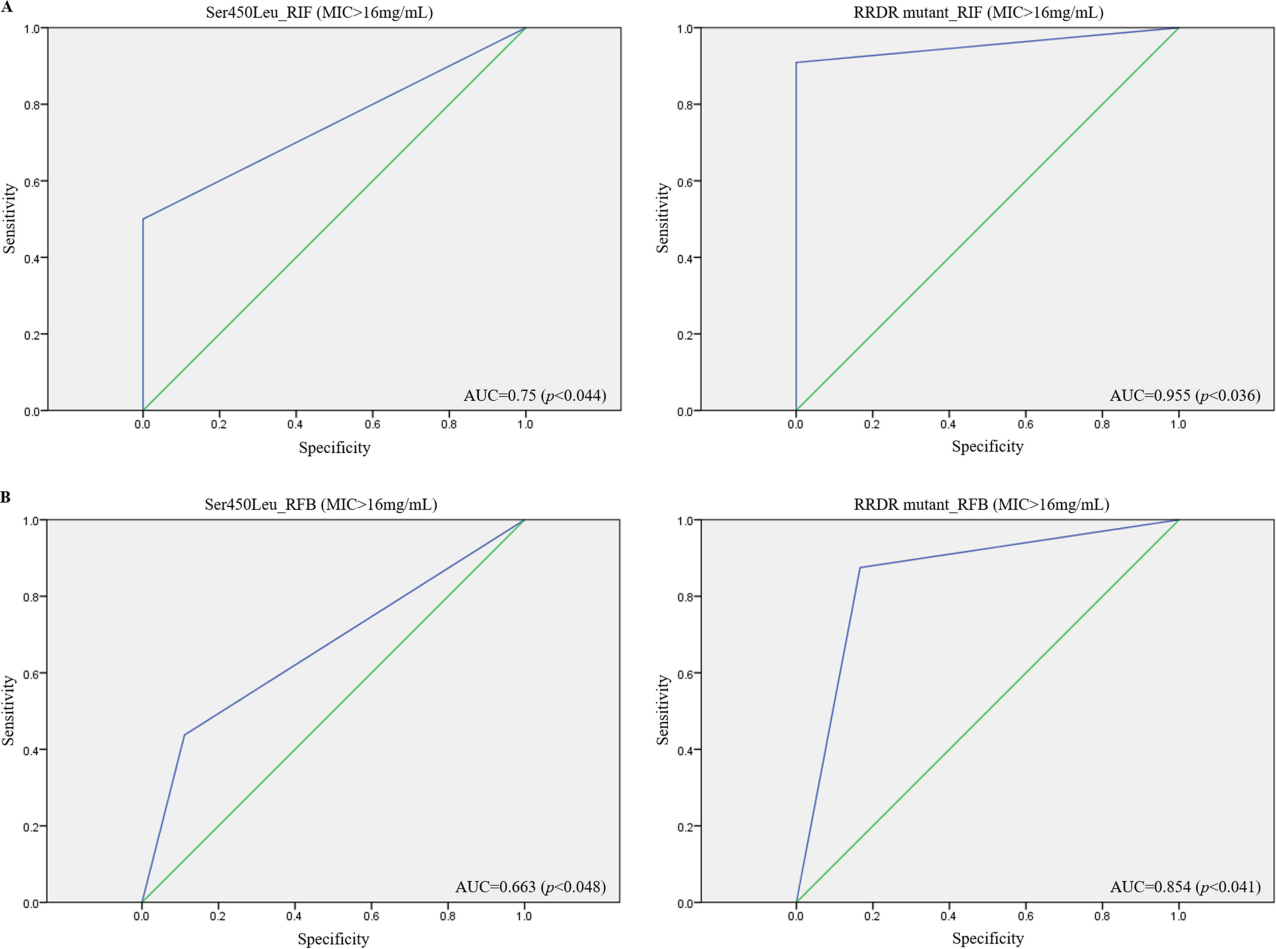
Predictive utility of whole-genome sequencing (WGS) assay results on the drug-resistant signatures of enrolled isolates estimated with statistical analyses. The utility of the presence of Ser450Leu or rifampin-resistance-determining region (RRDR)-variants for predicting (A) high RIF-resistant or (B) high RFB-resistant signatures of M. tuberculosis is evaluated using a receiver operating characteristic (ROC) analysis. MIC, minimum inhibitory concentration; AUC, area under the ROC curve.

The predictive utility of the high-confidence Ser450Leu and RRDR mutants for RIF resistance in clinical isolates was estimated using a receiver operating characteristic (ROC) curve analysis. The area under the curve (AUC) indicated the predictive efficacy of Ser450Leu (Fig. 2A, left panel; AUC = 0.75) and RRDR mutants, including Ser450Leu, Ser450Trp, His445Gln, and Gln432Lys, (Fig. 2A, right panel; AUC = 0.955) in classifying high RIF resistance (>16 μg/mL) in the enrolled isolates. Nevertheless, decreases in the predictive utility of Ser450Leu (Fig. 2B, left panel; AUC = 0.663) and RRDR mutants (Fig. 2B, right panel; AUC = 0.854) toward the high RFB-resistance signature (>8 μg/mL) were noted, which were potentially associated with the presence of emerging variants, including Phe669Val, Leu206Ile, and Met148Leu, identified in this study (Table 4, genotyping no. 7~10; RFB MIC < 0.5 μg/mL). These results suggested the predictive value of the WGS assay for precise diagnosis or treatment of RIF or RFB-resistant isolates.

## DISCUSSION

The turnaround time of a drug-susceptibility test (DST) or MIC assay for MTB is a critical issue regarding diagnostic efficacy and subsequent treatment (19). A high-throughput WGS approach coupled with a bioinformatics pipeline constitutes a potential strategy for overcoming this issue (20). Among current sequencing analyses, the ONT platform is practical for sequencing the GC-rich or repetitive proline-glutamate regions within the MTB genome (15, 21). Using the ONT sequencing pipeline, emerging variants within the MTB genome were identified and their additive or reductive influences on drug-resistance signature were subsequently demonstrated.

Rifabutin is a derivate of the rifamycin family which shares common properties with RIF (22). RIF or RFB resistance is predominantly related to mutations within the 81-bp RRDR of *rpoB* codons (codons 507~533 in E. coli and codons 426~452 in MTB) (18). Mutations at codons Asp435, His445, and Ser450 confer phenotypical resistance to over 90% of RIF- or RFB-resistant strains (23, 24). In contrast, variants at codons Leu430Prp, Asp435Gly/Tyr, Ser431Leu, His435Tyr/Leu/Asp/Asn, Ser450Gln, and Leu452Pro were demonstrated to be associated with RIF resistance but with phenotypic susceptibility to RFB (18, 23, 25). Moreover, the association of RIF resistance with the presence of an amino acid substitution outside the RRDR of the MTB genome, such as Val170Phe or Ile491Phe, has been demonstrated in previous studies (26). The presence of non-RRDR variants may interfere with the targeting strength of RIF or RFB to the mutant rpoB protein, which is worthy of further pursuit with corresponding assays. Using high-throughput sequencing, further evidence regarding the compensatory or resistance effects of emerging *rpoB* mutations on RIF or RFB resistance could be continuously provided.

Nucleic acid amplification-derived approaches, such as the Cepheid GeneXpert MTB/RIF assay, have been extensively used for diagnosing MTB infections and characterizing drug-resistance signatures (27). GeneXpert is a semiquantitative nested real-time PCR assay which can be used to diagnose suspected MDR-TB and/or HIV-infected patients (28). Although the Cepheid GeneXpert system was recommended by the WHO for diagnostic use in RIF resistance, the nature of the mutant *rpoB* gene has not been precisely pinpointed (27). In addition to the identification of high-confidence or emerging variants within the target gene, such as *rpoB*, establishment of genomic variant profiles using a WGS assay provides comprehensive information regarding lineage tracing and the evolutionary origins of MTB isolates (29, 30). A combination of WGS-generated genomic information and the drug-resistant profiles of MTB isolates could be applied to design treatment regimens and tailor public interventions toward diverse drug-resistant MTB isolates (31).

In this study, the presence of emerging variants within the *rpoB* gene was associated with differential resistance to RIF or RFB. In addition to the high-confidence group-1 Ser450Leu, Ser450Trp, and Gln432Lys variants, correlations between the emerging *rpoB* His445Gln or Asp531Gly variants and high RIF- or RFB-resistance were characterized by genotypic and phenotypic analyses in this study. Moreover, the existence of the novel Met148Leu, Leu206Ile, Phe669Val, and Leu443Trp variants within the *rpoB* gene shared influence on reducing RFB-resistance signatures. These results may influence treatment regimens for RIF- or RFB-resistant MTB isolates with further investigation. Nevertheless, these findings suggest the practical adoption of genotypic sequencing as an alternative strategy for the precise diagnosis and treatment of MTB patients.

## MATERIALS AND METHODS

### Study overview.

Whole-genome sequencing was conducted using the ONT long-read sequencing platform to identify nonsynonymous variants within the *rpoB* genes in RIF-susceptible and RIF-resistant MTB isolates.

### Ethics statement for sample collection.

Enrollment of anonymous clinical isolates was reviewed and approved by the Institutional Review Board of Taipei Medical University (approval no. N201912076). In this study, 30 RIF-susceptible and 20 RIF-resistant MTB isolates were enrolled from clinical specimens at Taipei Municipal Wan Fang Hospital.

### Susceptibility test.

Drug susceptible tests were conducted at the Department of Laboratory Medicine at Taipei Municipal Wan Fang Hospital using an agar proportion assay. In brief, suspensions with a turbidity of 1.0 McFarland standard were prepared from an MTB isolate inoculated on Lowenstein-Jensen medium. By examining the turbidity using a nephelometer, the suspension was subjected to the inoculum for all dilutions. Next, 100 μL of 10^−2^ and 10^−4^ dilutions of the standard inoculum was spread on 7H10 Agar with or without RIF. Drug resistance was determined as more than 1% colony growth in the presence of the drug compared to that in the absence of the drug.

### Extraction of high-molecular-weight genomic DNA.

A single colony of each MTB isolate was inoculated with 1 mL Middlebrook 7H9 Broth. The bacterial stock containing 30% glycerol was then preserved in −80°C freezer for following assay. MTB stocks were inoculated on Lowenstein-Jensen media in the absence of RIF. Multiple colonies in a single isolate were inactivated in 200 μL nuclease-free water at 95°C or 15 min. gDNA was extracted using a Presto Mini gDNA Bacteria kit (Geneaid, Taipei, Taiwan) according to the manufacturer’s instructions. The DNA concentration was determined using a Qubit fluorometer (Thermo Fisher Scientific, Wilmington, DE) with a fluorometric kit (GeneCopoeia, Rockville, MD).

### MIC test.

MIC assays were conducted using Sensititre MYCOTB MIC plates (Thermo Fisher Scientific) according to the manufacturer’s instructions. In brief, the MTB isolate was first subcultured on 7H10 Agar (Becton, Dickinson and Co., Sparks, MD). Multiple colonies were resuspended with glass beads in a saline-Tween solution, and the turbidity was adjusted to a McFarland standard of 0.5. A 100-μL volume of the resuspension was next mixed with 11 mL 7H9 broth containing oleic acid albumin-dextrose-catalase (Trek Diagnostic Systems, Cleveland, OH). A 100-μL volume of diluent was inoculated in each well of the MYCOTB plate and sealed with a permanent plastic seal at 37°C in 5% CO_2_. Plates were monitored on days 7, 10, 14, and 21 using a mirrored viewer. The lowest concentration with no visible growth was considered the MIC of rifampin or rifabutin.

### Whole-genome sequencing and variant identification.

Whole-genome sequencing of MTB gDNA was performed using the long read-sequencing approach (MinION, Oxford Nanopore Technologies [ONT], UK). In brief, 500 ng of gDNA was homogenized to 8,000-bp fragments using a g-TUBE device (Covaris, Woburn, MA) according to the manufacturer’s instructions. The fragmented gDNA was used for library construction using a Ligation Sequencing kit (SQK-LSK109; ONT) coupled with a Native Barcoding Expansion kit (EXP-NBD104 and 114; ONT) according to the manufacturer’s protocol. The barcoded library was captured, washed, and eluted from magnetic beads (AMPure XP, Beckman Coulter, High Wycombe, United Kingdom). Next, 0.7 μg of the pooled library was loaded and sequenced on the flow cells (FLO-MIN106D R9.4.1, ONT). The numbers of sequenced reads of each sample were 160,000~200,000 to reach a reading depth of 200. The quality and quantity of sequencing results were assessed using the EPI2ME website algorithm (https://epi2me.nanoporetech.com). The coverage rate of sequenced reads to the MTB reference was estimated by applying FastQ custom alignment (ONT). Analytical results of variant classification were aligned to the MTB reference (M. tuberculosis H37Rv, GenBank ID: NC_000962.3) using the bacterial small variant calling workflow, composed of the Medaka variant calling pipeline with long-read data sets via the EPI2ME Labs Launcher (ONT). The sequencing quality, reading depth, and variant calling with long-read sequencing results were synchronously assessed using the CLC genomics workbench (Qiagen v21.0.5; CLC bio, Aarhus, Denmark).

### Statistical analysis.

Experimental results were statistically analyzed using a one- or two-way analysis of variance (ANOVA) followed by Tukey’s multiple-comparison *post hoc* test. Analytical results are presented as the mean ± standard error of the mean (SEM) and considered significant at *P* values of <0.05 (*, *P* < 0.05; **, *P* < 0.01; ***, *P* < 0.005). The utility of identified variants for predicting RIF resistance was evaluated with the ROC curve and area under the ROC curve (AUC) ratio using SPSS Statistics 19 (IBM, Armonk, NY).

### Data availability.

The raw whole-genome sequencing data supporting the results of this article are available upon request.

## References

[1] WHO. 2020. Global tuberculosis report 2020. Available from https://www.who.int/publications/i/item/9789240013131. WHO, Geneva, Switzerland.

[2] WHO. 2018. Global tuberculosis report 2018. Available from https://apps.who.int/iris/bitstream/handle/10665/274453/9789241565646-eng.pdf. WHO, Geneva, Switzerland.

[3] Sharling L, Marks SM, Goodman M, Chorba T, Mase S. 2020. Rifampin-resistant tuberculosis in the United States, 1998–2014. Clin Infect Dis 70:1596–1605. doi:10.1093/cid/ciz491. 31233131 PMC6925655

[4] Li MC, Lu J, Lu Y, Xiao TY, Liu HC, Lin SQ, Xu D, Li GL, Zhao XQ, Liu ZG, Zhao LL, Wan KL. 2021. rpoB mutations and effects on rifampin resistance in *Mycobacterium tuberculosis*. Infect Drug Resist 14:4119–4128. doi:10.2147/IDR.S333433. 34675557 PMC8502021

[5] Zaw MT, Emran NA, Lin Z. 2018. Mutations inside rifampicin-resistance determining region of rpoB gene associated with rifampicin-resistance in *Mycobacterium tuberculosis*. J Infect Public Health 11:605–610. doi:10.1016/j.jiph.2018.04.005. 29706316

[6] Kumar S, Jena L. 2014. Understanding rifampicin resistance in tuberculosis through a computational approach. Genomics Inform 12:276–282. doi:10.5808/GI.2014.12.4.276. 25705170 PMC4330266

[7] Farhat MR, Sixsmith J, Calderon R, Hicks ND, Fortune SM, Murray M. 2019. Rifampicin and rifabutin resistance in 1003 *Mycobacterium tuberculosis* clinical isolates. J Antimicrob Chemother 74:1477–1483. doi:10.1093/jac/dkz048. 30793747 PMC6524487

[8] Opota O, Mazza-Stalder J, Greub G, Jaton K. 2019. The rapid molecular test Xpert MTB/RIF ultra: towards improved tuberculosis diagnosis and rifampicin resistance detection. Clin Microbiol Infect 25:1370–1376. doi:10.1016/j.cmi.2019.03.021. 30928564

[9] Ma P, Luo T, Ge L, Chen Z, Wang X, Zhao R, Liao W, Bao L. 2021. Compensatory effects of *M. tuberculosis* rpoB mutations outside the rifampicin resistance-determining region. Emerg Microbes Infect 10:743–752. doi:10.1080/22221751.2021.1908096. 33775224 PMC8057087

[10] Heupink TH, Verboven L, Warren RM, Van Rie A. 2021. Comprehensive and accurate genetic variant identification from contaminated and low-coverage *Mycobacterium tuberculosis* whole genome sequencing data. Microb Genom 7:000689. doi:10.1099/mgen.0.000689. 34793294 PMC8743552

[11] Peker N, Schuele L, Kok N, Terrazos M, Neuenschwander SM, de Beer J, Akkerman O, Peter S, Ramette A, Merker M, Niemann S, Couto N, Sinha B, Rossen JW. 2021. Evaluation of whole-genome sequence data analysis approaches for short- and long-read sequencing of *Mycobacterium tuberculosis*. Microb Genom 7:000695. doi:10.1099/mgen.0.000695. 34825880 PMC8743536

[12] Meehan CJ, Goig GA, Kohl TA, Verboven L, Dippenaar A, Ezewudo M, Farhat MR, Guthrie JL, Laukens K, Miotto P, Ofori-Anyinam B, Dreyer V, Supply P, Suresh A, Utpatel C, van Soolingen D, Zhou Y, Ashton PM, Brites D, Cabibbe AM, de Jong BC, de Vos M, Menardo F, Gagneux S, Gao Q, Heupink TH, Liu Q, Loiseau C, Rigouts L, Rodwell TC, Tagliani E, Walker TM, Warren RM, Zhao Y, Zignol M, Schito M, Gardy J, Cirillo DM, Niemann S, Comas I, Van Rie A. 2019. Whole genome sequencing of *Mycobacterium tuberculosis*: current standards and open issues. Nat Rev Microbiol 17:533–545. doi:10.1038/s41579-019-0214-5. 31209399

[13] Jain M, Olsen HE, Paten B, Akeson M. 2016. The Oxford Nanopore MinION: delivery of nanopore sequencing to the genomics community. Genome Biol 17:239. doi:10.1186/s13059-016-1103-0. 27887629 PMC5124260

[14] Votintseva AA, Bradley P, Pankhurst L, Del Ojo Elias C, Loose M, Nilgiriwala K, Chatterjee A, Smith EG, Sanderson N, Walker TM, Morgan MR, Wyllie DH, Walker AS, Peto TEA, Crook DW, Iqbal Z. 2017. Same-day diagnostic and surveillance data for tuberculosis via whole-genome sequencing of direct respiratory samples. J Clin Microbiol 55:1285–1298. doi:10.1128/JCM.02483-16. 28275074 PMC5405248

[15] Cervantes J, Yokobori N, Hong BY. 2020. Genetic identification and drug resistance characterization of *Mycobacterium tuberculosis* using a portable sequencing device. A pilot study. Antibiotics (Basel) 9:548. doi:10.3390/antibiotics9090548. 32867304 PMC7559383

[16] Oxford Nanopore Technologies. https://nanoporetech.com. Accessed 15 March. OST, Oxford, United Kingdom.

[17] WHO. 2021. Catalogue of mutations in *Mycobacterium tuberculosis* complex and their association with drug resistance. WHO, Geneva, Switzerland.

[18] Jamieson FB, Guthrie JL, Neemuchwala A, Lastovetska O, Melano RG, Mehaffy C. 2014. Profiling of rpoB mutations and MICs for rifampin and rifabutin in *Mycobacterium tuberculosis*. J Clin Microbiol 52:2157–2162. doi:10.1128/JCM.00691-14. 24740074 PMC4042728

[19] Huo F, Ma Y, Liu R, Ma L, Li S, Jiang G, Wang F, Shang Y, Dong L, Pang Y. 2020. Interpretation of discordant rifampicin susceptibility test results obtained using GeneXpert vs phenotypic drug susceptibility testing. Open Forum Infect Dis 7:ofaa279. doi:10.1093/ofid/ofaa279. 32766385 PMC7397830

[20] Daum LT, Konstantynovska OS, Solodiankin OS, Liashenko OO, Poteiko PI, Bolotin VI, Hrek II, Rohozhyn AV, Rodriguez JD, Fischer GW, Chambers JP, Gerilovych AP. 2018. Next-generation sequencing for characterizing drug resistance-conferring *Mycobacterium tuberculosis* genes from clinical isolates in the Ukraine. J Clin Microbiol 56:e00009-18. doi:10.1128/JCM.00009-18. 29563202 PMC5971536

[21] Chan WS, Au CH, Chung Y, Leung HCM, Ho DN, Wong EYL, Lam TW, Chan TL, Ma ESK, Tang BSF. 2020. Rapid and economical drug resistance profiling with Nanopore MinION for clinical specimens with low bacillary burden of *Mycobacterium tuberculosis*. BMC Res Notes 13:444. doi:10.1186/s13104-020-05287-9. 32948225 PMC7501614

[22] Sumandeep KG, George AG. 2011. Rifamycin inhibition of WT and Rif-resistant *Mycobacterium tuberculosis* and *Escherichia coli* RNA polymerases *in vitro*. Tuberculosis (Edinb) 91:361–369. doi:10.1016/j.tube.2011.05.002. 21704562

[23] Rukasha I, Said HM, Omar SV, Koornhof H, Dreyer AW, Musekiwa A, Moultrie H, Hoosen AA, Kaplan G, Fallows D, Ismail N. 2016. Correlation of rpoB mutations with minimal inhibitory concentration of rifampin and rifabutin in *Mycobacterium tuberculosis* in an HIV/AIDS endemic setting, South Africa. Front Microbiol 7:1947. doi:10.3389/fmicb.2016.01947. 27994580 PMC5136537

[24] Vadim M, Nathan TS, Maxwell AS, George AG, Katsuhiko SM. 2017. Structural basis for rifamycin resistance of bacterial RNA polymerase by the three most clinically important RpoB mutations found in *Mycobacterium tuberculosis*. Mol Microbiol 103:1034–1045. doi:10.1111/mmi.13606. 28009073 PMC5344776

[25] Berrada ZL, Lin SY, Rodwell TC, Nguyen D, Schecter GF, Pham L, Janda JM, Elmaraachli W, Catanzaro A, Desmond E. 2016. Rifabutin and rifampin resistance levels and associated rpoB mutations in clinical isolates of *Mycobacterium tuberculosis* complex. Diagn Microbiol Infect Dis 85:177–181. doi:10.1016/j.diagmicrobio.2016.01.019. 27036978 PMC4873381

[26] Jagielski T, Bakuła Z, Brzostek A, Minias A, Stachowiak R, Kalita J, Napiórkowska A, Augustynowicz-Kopeć E, Żaczek A, Vasiliauskiene E, Bielecki J, Dziadek J. 2018. Characterization of mutations conferring resistance to rifampin in *Mycobacterium tuberculosis* clinical strains. Antimicrob Agents Chemother 62:e01093-18. doi:10.1128/AAC.01093-18. 30061294 PMC6153850

[27] WHO. 2011. Automated real-time nucleic acid amplification technology for rapid and simultaneous detection of tuberculosis and rifampicin resistance: Xpert MTB/RIF system. Policy statement. Available from https://apps.who.int/iris/handle/10665/44586. WHO, Geneva, Switzerland. 26158191

[28] Pallas SW, Courey M, Hy C, Killam WP, Warren D, Moore B. 2018. Cost analysis of tuberculosis diagnosis in Cambodia with and without Xpert(R) MTB/RIF for people living with HIV/AIDS and people with presumptive multidrug-resistant tuberculosis. Appl Health Econ Health Policy 16:537–548. doi:10.1007/s40258-018-0397-3. 29862440 PMC6050005

[29] Anirvan C, Kayzad N, Dhananjaya S, Camilla R, Nerges M. 2017. Whole genome sequencing of clinical strains of *Mycobacterium tuberculosis* from Mumbai, India: a potential tool for determining drug-resistance and strain lineage. Tuberculosis (Edinb) 107:63–72. doi:10.1016/j.tube.2017.08.002. 29050774

[30] Takiff HE, Feo O. 2015. Clinical value of whole-genome sequencing of *Mycobacterium tuberculosis*. Lancet Infect Dis 15:1077–1090. doi:10.1016/S1473-3099(15)00071-7. 26277037

[31] He W, Tan Y, Liu C, Wang Y, He P, Song Z, Liu D, Zheng H, Ma A, Zhao B, Ou X, Xia H, Wang S, Zhao Y. 2022. Drug-resistant characteristics, genetic diversity, and transmission dynamics of rifampicin-resistant *Mycobacterium tuberculosis* in Hunan, China, revealed by whole-genome sequencing. Microbiol Spectr 10:e0154321. doi:10.1128/spectrum.01543-21. 35171016 PMC8849054

